# Angiographically Silent Ruptured Dural Arteriovenous Fistula Presenting As Subdural Hematoma

**DOI:** 10.7759/cureus.31204

**Published:** 2022-11-07

**Authors:** Zachary A Abecassis, Guilherme Barros, Laligam N Sekhar, Randall M Chesnut

**Affiliations:** 1 Neurological Surgery, University of Washington, Seattle, USA

**Keywords:** case report, vascular malformation, davf, subdural hematoma, arteriovenous fistula

## Abstract

Dural arteriovenous fistulas (dAVF) are aberrant vascular communications that can have devastating effects ranging from headaches to death. Typically, these malformations are identifiable on a CT angiogram (CTA) and confirmed via catheter angiography. We present a case of a female patient who presented with a headache and was found to have a large holohemispheric subdural hematoma. Given the lack of trauma, a CTA was performed. The CTA revealed abnormal vessels in the anterior temporal lobe spanning her hematoma. A diagnostic cerebral angiogram was performed; no early venous drainage was detected. When the patient was taken to the operating room for subdural hematoma evacuation, an aberrant connection from a superficial cortical vein to the middle meningeal artery was identified and ligated. Although rare, this case demonstrates that patients can present with ruptured vascular malformations that are radiographically silent on cerebral angiography.

## Introduction

Dural arteriovenous fistulas (dAVF) are well-described malformations that represent 15% of intracranial arteriovenous shunts [1]. These malformations typically present with a combination of intracranial hemorrhage and subarachnoid hemorrhage. Some variation in clinical presentation is observed, depending on the presence or absence of a flow-related aneurysm [2]. Typically, these malformations are identifiable on a CT angiogram (CTA) and confirmed via catheter angiography. Multiple cases have been reported of patients with a ruptured dAVF presenting with a spontaneous subdural hematoma and intraparenchymal hemorrhage. However, all of these cases were confirmed with cerebral angiography [3-8]. Here, we present a case of a young woman with a traumatic large holohemispheric subdural hematoma with suspicion for a dAVF on CTA that was negative on cerebral angiography but with the pathology confirmed intraoperatively.

## Case presentation

**Figure 1:**
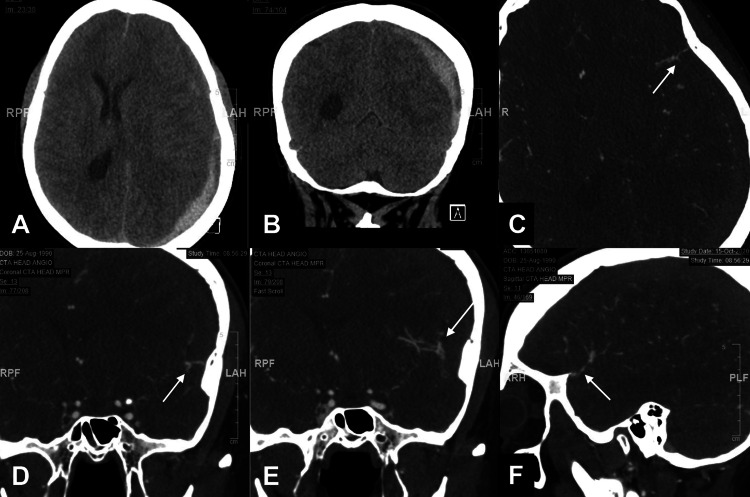
Non-contrast CT and CT angiogram images. A: Non-contrast axial and B: Coronal head CT, approximately seven hours after initial imaging, demonstrating a left holohemispheric, mixed density subdural hematoma with layering hyperdense blood products and approximately 1.1 cm of midline shift. CTA showing C: Axial, D: Coronal, E: Coronal, slightly more posterior compared with D; and F: Sagittal, capturing a prominent superficial cortical vasculature crossing over the hematoma as a potential etiology for the spontaneous subdural hemorrhage. White arrows highlighting location of presumed vascular abnormality.

A 30-year-old female patient initially presented to an outside hospital complaining of a sudden-onset, severe headache. Given a history of migraines, she was treated with rescue medications and discharged home. Several days later, she presented to our institution’s ED with progressive worsening of her headache along with new-onset lethargy and photophobia. The patient was lethargic but easily arousable, following commands and answering questions appropriately upon evaluation. She had no precedent trauma or known cerebrovascular malformations. She was not taking any anticoagulant or antiplatelet medications. She also had no family history of intracranial hemorrhages or aneurysms. Initial head non-contrast CT showed a left-sided mixed density acute on chronic left-sided subdural hematoma with approximately 1 cm left-to-right midline shift (Figures 1A-1B). There was an associated local mass effect with effacement of the left lateral ventricle and asymmetric dilatation of the right lateral ventricle suggesting entrapment (Figure 1A). These findings were stable on a repeat CT head seven hours later. Due to the lack of trauma history, a head and neck CTA was obtained, which showed prominent superficial vasculature along the anterior left frontal and temporal lobe adjacent to the subdural hematoma (Figures 1C-1F). 

**Figure 2:**
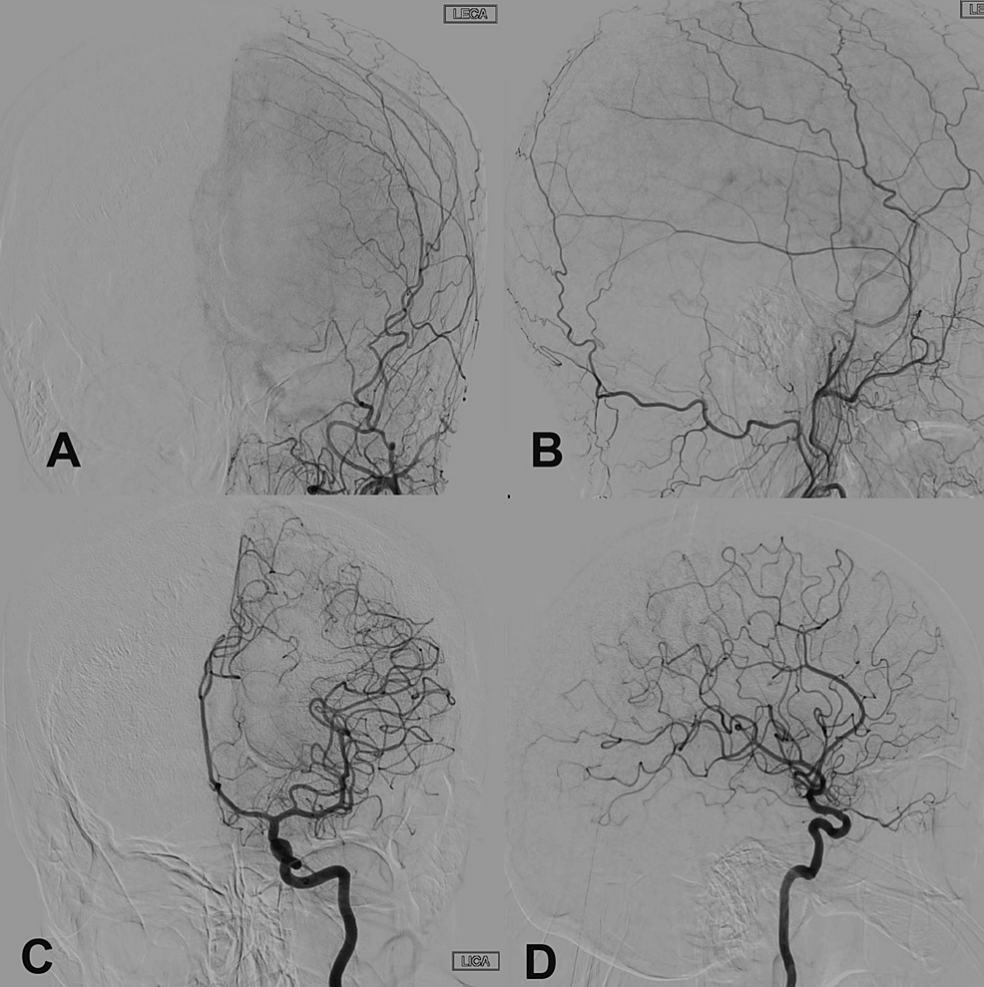
Diagnostic cerebral angiograms. Diagnostic cerebral angiogram of the left external carotid artery. A: Anteroposterior and B: Lateral. Diagnostic cerebral angiogram of the internal carotid artery. C: Anteroposterior, and D: Lateral. Images demonstrate no obvious vascular abnormalities or early venous drainage.

This finding prompted the urgent acquisition of a diagnostic catheter angiogram to assess for vascular malformation. However, the angiogram did not show any evidence of early venous drainage to suggest a ruptured arteriovenous malformation (AVM) or dAVF (Figures 2A-2D).

**Figure 3:**
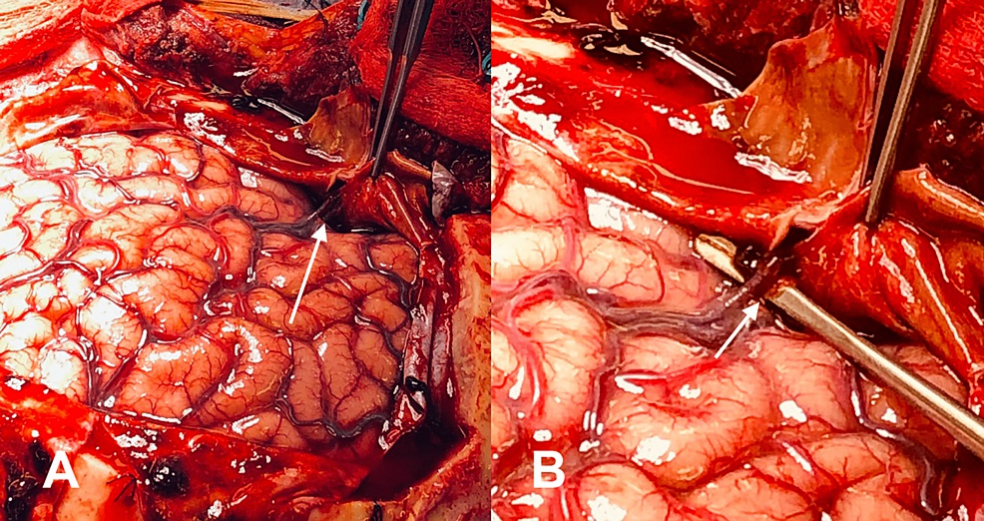
Intraoperative pictures of an abnormal bridging vein with fistulous connection to the middle meningeal artery. A: Gently retracting dura to show abnormal connection. B: Highlighting aberrant vessel prior to obliteration. White arrows show abnormal bridging vessel.

Given the mass effect of the patient’s subdural hematoma and declining mental status, the patient was taken to the operating room for evacuation after her catheter angiogram. We placed the patient in Mayfield skull pins and used a Kempe incision for a large frontotemporoparietal craniotomy [9]. After elevation of the skull flap, we opened the dura in a stellate fashion, and the subdural hematoma immediately egressed under pressure. The subdural hematoma was of mixed density, with both solid dark clots and dark brown fluid. After the adequate evacuation of the hematoma, we could see several hemorrhagic petechiae throughout the cerebral cortical surface. In addition, we identified large pulsatile veins in the anterior portion of the Sylvian fissure, with a direct connection to the dura just posterior and lateral to the greater sphenoid wing. The middle meningeal artery (MMA) appeared to be feeding directly into these superficial veins in the Sylvian fissure through the dura (Figures 3A-3B). 

**Figure 4:**
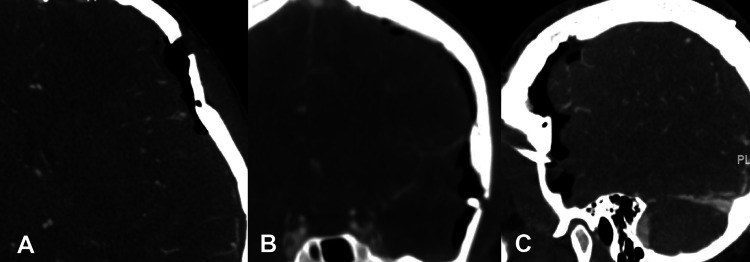
Post-operative CT angiogram images. A: Axial; B: Coronal; and C: Sagittal images, demonstrating no evidence of the bridging vessels seen in pre-operative imaging.

We cauterized the MMA artery, the draining veins, and the dura at that connection point and then cut the coagulated veins. Subsequently, we observed immediately decreased pulsatility of the cerebral veins within the Sylvian fissure. A postoperative head CTA showed appropriate evacuation of the subdural hematoma and no further evidence of persistent arteriovenous fistula (Figures 4A-4C).

The patient was discharged home on a postoperative day three without neurologic deficits. She returned to our clinic six weeks later, neurologically intact, reporting only mild daily headaches and some scalp tenderness. Her follow-up head CT showed a small left hygroma underlying the craniotomy without evidence of recurrent subdural hematoma.

## Discussion

Here we present a case of a dAVF fed by the MMA and presenting as an isolated subdural hematoma without angiographic evidence of early venous drainage. Given the presentation of a young patient with a spontaneous subdural hematoma and CTA evidence of aberrant vessels, an underlying cerebrovascular malformation was suspected as the most likely etiology. However, after diagnostic cerebral angiography did not reveal any fistula or malformation, the patient was taken to the operating room for evacuation and exploration. There have been multiple cases reported of patients with a ruptured dAVF presenting with a spontaneous subdural hematoma [3-6], and in two case reports, an associated intraparenchymal hematoma [7, 8]. However, all of these case reports had dAVF confirmed radiographically by a cerebral angiogram showing early venous drainage. A ruptured aneurysm can rarely present with isolated subdural bleeding, especially aneurysms outside the circle of Willis, adherent aneurysms, or aneurysms with a rupture that disrupts the pia-arachnoid membrane at anatomically vulnerable locations [10]. All these examples should be considered when atraumatic subdural hematomas are present and CTA should be strongly considered.

There is no conventional clinical presentation for patients with dAVF presenting with a subdural hematoma. In one instance, the patient had a similar presentation to our patient with a sudden onset headache and syncope [3]. Other presentations were different, with one patient presenting with seizures [5] and another with progressive headaches over a few days [6]. All of these cases were eventually treated with surgical ligation; however, all three patients underwent endovascular embolization prior to ligation. In this report, the patient’s pathology was angiographically silent, making endovascular intervention impossible. Though this case deviates in some ways from other reported cases, it is essential to keep a vascular malformation on the differential for a non-traumatic intracranial bleed.

To the best of our knowledge, this is the first case report where cerebral angiography was negative for early venous drainage, resulting in the identification of the dAVF only intraoperatively under direct visualization. We suspect the draining veins of the dAVF may have been partially thrombosed after the rupture and formation of the subdural hematoma. This may be why the catheter angiogram showed no signs of early venous drainage suggestive of a dAVF. However, intraoperatively during subdural hematoma evacuation, the fistulous connection was clearly pulsatile and at least partially patent, making ligation necessary. As described, our surgical management was different from other reported cases, mainly because the lesion appeared angiographically silent on diagnostic cerebral angiogram.

## Conclusions

This case illustrates the importance of neurosurgeons, neurologists, ER physicians, and primary care physicians recognizing cerebrovascular malformations as the likely etiology of spontaneous intracranial hemorrhages, including subdural hematomas. Despite a potentially negative catheter angiogram in these patients, neurosurgeons need to be prepared to identify and ligate a potential dAVF in the operating room during evacuation.
